# Mesenchymal stromal cell infusions of umbilical cord-derived mesenchymal stromal cells in children with Recessive Dystrophic Epidermolysis Bullosa (MissionEB): a qualitative sub study of a randomised, double-blind, placebo controlled, crossover, phase 3 trial

**DOI:** 10.1186/s13023-026-04350-1

**Published:** 2026-06-03

**Authors:** Katie Biggs, Shamila Ditta, Maria L. Bageta, Pablo Lopez-Balboa, Rachel Glover, Kate Hutchence, Diana Papaioannou, Steven Julious, Cindy Cooper, Gabriela Petrof, Anna E. Martinez

**Affiliations:** 1 Clinical Trials Research Unit (CTRU), Sheffield Centre for Health and Related Research (SCHARR), School of Medicine and Population Health, University of Sheffield, Sheffield, UK; 2 Dermatology Department, Great Ormond Street Hospital NHS Foundation Trust, London, UK

**Keywords:** Recessive Dystrophic Epidermolysis Bullosa (RDEB), Quality of life, Randomised controlled trial, Cross-over study, Double blind, Qualitative, Cell therapy, Mesenchymal stem cells

## Abstract

**Background:**

Recessive Dystrophic Epidermolysis Bullosa (RDEB) is a rare genetic skin condition causing fragile skin, blistering, and scarring. It leads to chronic pain, slow wound healing, and severe limitations, profoundly impacting patient and family quality of life. Umbilical cord tissue-derived mesenchymal stromal cells (UC-MSC) have shown therapeutic promise. The Mission EB trial (ISRCTN14409785; registration date 25/03/2021) assessed UC-MSC safety and effectiveness in children with RDEB in a placebo-controlled, double-blinded, crossover study.

**Methods:**

This study used a qualitative research design with semi-structured interviews and thematic analysis, to explore Mission EB trial treatment impact on the quality of life of patients and parents. Parents and patients were interviewed at two time points (approximately 3- and 12-months post-randomisation). Purposive sampling included 10 parents and 6 children; 13 individuals (8 adults, 5 children) were interviewed twice (once in each study period). Interviews were transcribed verbatim, independently coded, with overall impressions agreed upon prior to unblinding.

**Results:**

RDEB significantly impacted daily life, marked by pain and itch. Participants were hopeful of the trial, willing to pursue minor improvements. UC-MSC infusions led to reduced pain/itchiness, improved wound healing, and resulted in fewer self-reported dressing changes. These benefits often translated to increased energy, improved eating, and greater daily activity participation. Benefits were more pronounced with active treatment. Negative effects were minimal, primarily venous access difficulties. Blinded participants could discern active UC-MSC from placebo based on symptom changes; 10 of 13 showed clear differences aligning with treatment. All parents interviewed expressed willingness for their child to receive treatment again.

**Conclusions:**

This qualitative research provides valuable insights into perceived benefits of UC-MSC treatment for children with RDEB. Interview findings regarding symptom improvement and participants’ ability to discern active treatment shed light on its perceived benefit in RDEB, especially in milder RDEB patients. The study highlights the critical importance of qualitative methodologies in adding to quantitative trial outcomes and capturing the meaningful impact of interventions on the lives of patients and families particularly where quantitative measures may not fully reflect lived experience.

**Supplementary Information:**

The online version contains supplementary material available at 10.1186/s13023-026-04350-1.

## Background

### Recessive Dystrophic Epidermolysis Bullosa (RDEB)

Epidermolysis Bullosa (EB) is a rare genetic skin condition that causes fragile skin, blistering, and scarring and patients experience long-term physical, social and economic consequences [1]. A severe subtype called Recessive Dystrophic EB (RDEB) is caused by mutations in the type VII collagen gene and leads to skin blisters after minor trauma, slow wound healing and can lead to fibrosis, limb contractures, and an increased risk of developing squamous cell carcinoma where there are chronic wounds and scarring [2, 3]. RDEB has no cure and treatment focuses on managing the symptoms, which can be painful [4] and time-consuming [5]; quality of life in patients with RDEB is worse than for other forms of EB [6]. Patients with RDEB (and EB) have severe limitations in function and social activities, a significant reduction in their quality of life [4, 7], in quality of life for their families and carers and can involve a high emotional and economic burden [8, 9].

The management of symptoms requires a multidisciplinary approach [4], and in recent years there has been a global effort to find effective treatments for RDEB symptoms, with cell-based therapies identified as potential effective treatments [10–12]. In an uncontrolled clinical trial, ‘mesenchymal stromal cells’ (MSCs) were given to children with RDEB and showed promising results, improving wound healing, and reducing discomfort [13]. While bone marrow-derived MSCs were used in that trial, there is evidence that umbilical cord tissue-derived MSCs (UC-MSCs) might be even more effective [14].

### Mission EB trial

The Mission EB trial (ISRCTN14409785) was a placebo controlled, double blinded, crossover trial looking at whether UC-MSCs (CORDStrom™, manufactured by INmune Bio) were safe and effective at treating children with RDEB [15]. To assess the effectiveness of UC-MSCs, participants received infusions at two timepoints, one active and one placebo. Participants were randomised (*N* = 37) to the order in which these were received, with patients receiving the active and placebo infusions nine months apart; 34 participants received at least one infusion. As this is a trial in a rare disease, and the sample size is small, we did not expect there to be statistically significant results. The quantitative results raised no safety concerns and although there were no changes in the primary outcome measure, the Epidermolysis Bullosa Disease Activity and Scarring Index (EBDASI), there were trends towards clinically meaningful results, with the most promising results in RDEB-intermediate patients [16].

In addition to evaluating quantitative outcomes, it is important to examine the impact of the treatment on the children and on their parent/guardian to add context to some of the outcomes this is particularly true in RDEB where there are no robust validated outcome measures [17, 18]. In the previous uncontrolled trial of MSCs for RDEB parents and children reported several benefits to treatment that were not picked up in the quantitative measures, such as better sleep, impact on family relationships and leisure time and reduction in work absences for the parents [13]. For these reasons, and as recommended to complement outcomes in rare diseases [19], a qualitative sub-study was planned from the outset to complement the crossover trial.

Previous qualitative research has demonstrated the impact EB can have on the whole family unit [20], that there is emotional turmoil experienced by carers [21], there is an impact on school, family, societal interactions, and daily life [22]).

### Purpose of current study

An interview study was conducted to explore the impact of the Mission EB trial treatment on patients’ and parents’ quality of life and patients’ symptoms. It was hoped these results would complement the quantitative results from the clinical trial by capturing the lived experience of the patients beyond that assessed in the trial outcomes.

## Methods

### Study design and setting

A qualitative research design was used in the form of semi-structured interviews using thematic analysis, guided by an interpretative theoretical approach [23] to understand participants experiences and perspectives in their own words. We interviewed parents and patients (children and young people over 6 years of age) at two time points during their involvement in the Mission EB trial (3- and 12-month follow-up points). Interviews were conducted either in person, at the participating hospitals: Birmingham Children’s Hospital (BCH) or Great Ormond Street Hospital (GOSH), in participants homes, or online via Google Meet (with or without video) according to the participants’ preference.

### Research team

KB designed and led the qualitative research with input from the Mission EB team who helped to develop the topic guide and interpret the results. A research assistant (SD, female, British Pakistani, BSc, MA) conducted all interviews, undertaking the research as part of their employment, and trained by KB. SD was not known to the participants prior to the first interview and did not have lived experience of the condition or any assumptions about the trial treatment. Interviewing participants at two timepoints (aligned with the trial crossover period) allowed for comparative reflexive analysis of the participants’ real time changes rather than relying on a single snapshot.

### Sampling strategy

Purposive sampling was used, and we aimed to recruit 10 pairs consisting of a patient and their parent/carer, split across the two sites, with a range of ages and severity. We did not account for data saturation as we were limited in numbers due to RDEB being a rare disease. Interview participants had already given consent to take part in the main trial. Due to the timing of the qualitative project, we were not able to interview the first ten participants in the trial as they had passed their 3-month time point once approvals had been gained, no participant refused to take part, but not all were invited due to timing and scheduling reasons (e.g. the interviewer could not travel to the trial site to undertake the interview at the same time as the patient; follow up visits were sometimes postponed or were not in the planned follow up window). Participants were informed of the objectives of the qualitative study through the information sheet.

### Data collection methods, processing and analysis

A semi-structured interview guide was developed based on discussions with the research team and patient representatives. The topic guide (Additional File 1) included questions about the impact of RDEB on the interviewees’ lives (and their children/families in the case of parents), and the impact of the trial treatment on the patients’ symptoms of RDEB and their lives. The topic guide was not piloted due to the small population reducing the sample, and to reduce unnecessary burden on participants. The clinical teams reviewed the questions and field notes were taken and following the first few interviews, with some prompts added based on important factors raised by participants, such as questions about bowel movements and energy levels. Interviews were conducted around the 3-month follow-up period and repeated at the 12-month follow-up time point. Interviewees were interviewed individually (but the child or parent were usually both present in the room), except for two interviews where both parents were interviewed together. We did not ask interviewees to check transcripts or to give feedback on the interviews.

All data was collected via interviews, transcribed verbatim and all interviews were coded by KB and SD independently in Nvivo14 software [24] using thematic analysis. KB and SD iteratively discussed the codes following the coding of a few interviews at a time and a coding tree was developed (Additional File 2).

As RDEB is a complex condition, with patients experiencing differences and changes in their symptoms, KB and SD also independently rated each interview as positive, negative or no change based on the overall responses from the participants, and this was discussed to produce an overarching view from each interview; we did not quantify the agreement/disagreement but there were no disagreements in the overall impression. This was undertaken prior to the researchers being unblinded to the intervention arm at each time point. Following discussion and agreement on the overarching findings, KB was unblinded to report the findings to the oversight committees and the funder along with the analysis of the 3-month follow-up data; the overarching view was not amended following this unblinding.

## Results

### Interview participants

Ten parents and six children agreed to be interviewed (four children were too young to be interviewed); 13 individuals (8 adults and 5 children) were interviewed at both time points. Interviews ranged from 4:06 min to 42:36 min (Additional File 3). Table 1 shows the baseline characteristics for the interviewed and the trial participants. We did not collect demographic data from parents, though we interviewed one father and nine mothers (with two additional fathers present in two interviews). Patients included a range of ethnicities, and a spread across sex and ages, (mean age of patient in the recruited pairs = 7.8 (0–15) and mean age of those patients interviewed = 11.8 [7–15]), and there were five pairs included from each of the two participating sites.

The interviewed population (*N* = 10) and the total trial population (*N* = 30) shared similar age profiles, with both groups having a median age of 8 years, and only a 10% difference in the number in each age group. There was a difference found in sex distribution, as the trial population was evenly split (50% male, 50% female), whereas the interviewed group was predominantly male (70%) and both groups were similar in ethnicity (predominantly White).

Regarding disease characteristics, and RDEB sub-type, with the majority in both cohorts RDEB Intermediate. However, the trial population generally presented with higher baseline EB measures than the interviewed subset. For example, the median overall EBDASI score was 146 for trial participants compared to 115 for those interviewed, and the trial group also showed higher mean scores for disease activity, damage, and skin involvement.

**Table 1:** Participant (patients’) baseline measures

Baseline demographics	Interviewed participants (*N* = 10)	Trial participants (mITT, *N* = 30)
Age at consent (years, grouped)		
< 10	6 (60%)	21 (70%)
>=10	4 (40%)	9 (30%)
Age at consent (years)		
N (%)	10 (100%)	30 (100%)
Mean (SD)	7.8 (5.8)	7.7 (4.8)
Median (IQR)	8 (3, 13)	8 (4, 13)
Min, Max	0, 15	0, 15
Sex		
Male	7 (70%)	15 (50%)
Female	3 (30%)	15 (50%)
Ethnicity		
White	6 (60%)	19 (63%)
Asian/Asian British	3 (30%)	6 (20%)
Mixed/multiple ethnic groups	1 (10%)	2 (7%)
Black/African/Caribbean/Black British	0 (0%)	1 (3%)
Other ethnic group	0 (0%)	2 (7%)
**Baseline EB measures**	**Total**	
RDEB sub-type		
Intermediate	6 (60%)	16 (53%)
Severe	4 (40%)	14 (47%)
Overall EBDASI Score		
N (%)	10 (100%)	30 (100%)
Mean (SD)	112.3 (64.6)	130.3 (62.0)
Median (IQR)	115 (77, 166)	146 (82, 178)
Min, Max	13, 208	13, 208
Activity Score		
N (%)	10 (100%)	30 (100%)
Mean (SD)	39.7 (29.1)	45.0 (24.2)
Median (IQR)	34 (15, 52)	45 (26, 60)
Min, Max	4, 86	4, 86
Damage Score		
N (%)	10 (100%)	30 (100%)
Mean (SD)	72.6 (39.6)	85.3 (41.7)
Median (IQR)	76 (51, 103)	102 (51, 117)
Min, Max	9, 122	9, 138
Skin score		
N (%)	10 (100%)	30 (100%)
Mean (SD)	50.0 (24.3)	57.6 (24.2)
Median (IQR)	58 (47, 66)	62 (38, 76)
Min., Max.	7, 76	7, 101

Outputs produced in STATA 19.5. The upper and lower quartile for all participants (mITT) can differ slightly from those in the published report [16]. Demographic tables in the published report were produced using R which by default uses a different calculation for quartiles

mITT = modified Intention To Treat (analysis population); SD = Standard Deviation; IQR = Interquartile Range

### Interview findings

Interviews were coded according to the symptoms and activities discussed, and the participants’ views on treatment and their effects. The final coding tree is provided in Additional File 2, where themes were around RDEB symptoms and life with RDEB as well as themes relating to the impact of the treatment, with some double coding to link the impact to specific symptoms. The findings are reported below, organised by the themes, starting with the impact of RDEB on their life in general, RDEB symptoms, impact on family life, the interviewees expectations of the Mission EB trial treatment, the impact of treatment on their symptoms and life, ending with comments on the trial. Quotes are presented in the text and in Table 2 - Themes and illustrative quotes regarding positive effects of active treatment on symptoms of RDEB. Table 3 presents the researchers’ (KB and SD) overarching impression from the interviews and includes the allocation, RDEB severity, order of infusions.

#### Living with RDEB

When describing what life is like with RDEB, their typical daily routine, parents and children often discussed how living with RDEB was at the core of their day-to-day life and that dealing with symptoms is often incorporated into daily activities that would be typical of any child for example, school.

*Normal for us really*,* but obviously is isn’t because if you were to look at a child at [NAME 1’s] age*,* compared to [NAME 2] it’s completely different because obviously get up in the morning*,* baths*,* dressing changes*,* medication*,* off to school*,* normally a couple of phones calls in the day and then come home*,* food*,* dressing change well if need be we pop blisters have a little wound check.* Parent F (RDEB Intermediate).

##### Impact of RDEB on the patients’ symptoms

The most common symptoms discussed were pain, wounds and itching, and the impact of these things on dressings, eating and going to the toilet. The children interviewed were generally accepting of their condition, and some expressed that they did not know any different. Parents reported being impressed by their child’s resilience, and ability to cope with RDEB and the symptoms, particularly pain.

*I think the worst thing for me is the pain because the pain affects everything in general so like with sleeping there’s pain. That’s annoying with sleep*,* with the dressing changes it’s the pain that’s annoying and the dressing change. With the itch after you’ve itched you have pain.* Patient D (RDEB Severe).

##### Impact of RDEB on the patients’ life and daily activities

The condition can also impact on the ability to do things at the weekends or attendance at school. It seemed that symptoms varied in their impact on the child; for e.g. ‘flare ups’ limited daily activities due to an increased impact of symptoms.

The impact on school and education was often referenced, with schools adapting to cater to the child’s specific needs. Whilst school was a key part of daily routine, parents and children discussed the ongoing challenges with participating in school activities, such as being able to join in with PE, or playing with peers.

*sometimes [they sit] in [their] wheelchair*,* and because it’s really cold they don’t even take [them] out*,* [they stay] inside class.* Parent J (RDEB Severe).

##### Impact of RDEB on the family life

Parents discuss the impact on their own lives, needing to be ‘on-call’ during the night and when doing activities and checking their child’s skin and dressings regularly. Parents expressed distress and upset when their child is particularly unwell or suffering with their symptoms.

*… it actually affects me a lot mentally so you know when just imagine your child crying screaming with pain and you can’t do anything*,* that literally*,* apart from breaking your heart it’s actually stressful.* Parent D (RDEB Severe).

Parents also discussed how play between siblings can be impacted by EB as siblings have had to adapt the way in which they play to prevent pain.

#### Mission EB treatment

##### Interviewees expectations of treatment

Parents and children all reported being hopeful about the trial treatment, and parents reported that they would do anything they could to help their child, even if it only helped a little bit.

*I’m happy to come whenever they call us for the trial because that one per cent is a lot I know maybe it’s nothing for somebody but it’s a lot for [them] *,* one per cent less pain for [them] one per cent real life how [they're] spending now it’s a lot.* Parent D (RDEB Severe).

##### Impact of UC-MSC treatment

Table 2 provides illustrative quotes around the impact of the treatment on symptoms and daily life from the treatment, with some additional quotes in the text illustrating the themes from the interviews. Quotes include the time point, where 3 m is the first period, and 12 m the second, with the order of treatment provided in brackets.

##### Positive impacts on symptoms

Positive impacts of an infusion reported were around reduction of pain and itchiness and improved wound healing and reduced the number of dressing changes needed.

*I think for like a good few weeks*,* like it actually did stop. I felt like way different and like I could just tell because If I was like on [a] normal day*,* I would be putting my bandages on and my socks on*,* then my shoes. I just feel itchiness and I would feel hurt*,* like it would hurt a little and feel the pain. But when I got the infusion done after that it would just feel normal*,* like how I would expect someone to feel if they didn’t have EB* Patient C, 3 m (RDEB Intermediate, UC-MSCs/Placebo).

Some parents reported positive impacts on their child’s energy levels since an infusion, or of improved recovery from other illnesses. Improvement in wound healing and pain were also reported to have a positive impact on the child’s eating.

*Everything really*,* so energy has been fantastic*,* health has been great [they haven’t] stopped, well it’s hard to tell [they have] got food stuck but it hasn’t supposed had a massive impact like it had before* Parent B, 3 m (RDEB Intermediate, UC-MSCs/Placebo).

Although there were more statements about improvement of symptoms when people had received the active treatment, there were some positive impacts reported in a few interviews where the patient had received placebo.

*I have been*,* I’ve been like what’s it called my friend always like comes knocking or saying do you want to play out and I normally say no because I am too tired but now*,* I can actually go outside and play as well.* Patient E, 3 m (RDEB Intermediate, Placebo/UC-MSCs).

##### Positive impacts on daily activities

The improvement in symptoms often led to positive impacts on daily activities. Below are quotes from patients and parents that had received the treatment approximately 3 months prior to the interview.

**Table 2:** Themes and illustrative quotes regarding positive effects of active treatment on symptoms of RDEB

Theme	Illustrative quotes
Pain	*“Normally I would like it would hurt when I have a bath but lately it hasn’t been hurting.”* Patient E, 12 m (RDEB Intermediate, Placebo/UC-MSCs) *“I do think things have become a little bit easier for [them] you know when [they were] waking up less and also [their] pain being less.”* Parent J, 12 m (RDEB Severe, Placebo/UC-MSC)
Itch	*“I was*,* I don’t know it made me feel more comfortable in my own skin it was less itchy*,* it was like and like my hands were less hot and when I like hot*,* it spreads then I itch. That was not happening that much.”* Patient C, 3 m (RDEB Intermediate, UC-MSCs/Placebo) *“[They sleep] better so obviously [they do] have an overnight feed so [they need their]bathroom more but overall [their] sleep is better [they don’t] seem as itchy that is one thing I’ve noticed [they don't] seem to itch as much as [they] used to do.”* Parent E, 12 m (RDEB Intermediate, Placebo/UC-MSCs)
Skin and wounds	*“Even when [they have] blisters I never really found [them] in pain in or itchiness or anything like that but I think it’s improved the quality of life because if we don’t have so many blisters we don’t have as many open wounds so I think that will make [them] feel a little bit more comfortable and even for us of course.”* Parent A, 3 m (RDEB Intermediate, UC-MSC/Placebo) *“Yeah the childminder said [their] skin is much better and I say well you don’t see the feet but maybe it’s true …… so we went to the hospital before Easter and [they were] vomiting and things and when the doctor arrived he has eczema and I say no it’s EB and he says the face it’s not the same as EB.”* Parent I, 12 m (RDEB Severe, Placebo/UC-MSC)
Sleep	*“Definitely*,* psychologically at least it’s reassuring to know that [they’re] not in pain and [they’re] acting and [they’re] sleeping as normal as possible I would say and that makes all the difference for me and dad as well.”* Parent A, 12 m (RDEB Intermediate, UC-MSCs/Placebo) *“Yeah because well yeah because when [they sleep] well I sleep well I don’t feel restless during the day”* Parent H, 12 m (RDEB Severe, Placebo/UC-MSCs)
Quality of Life	*“So the benefits I’ve seen [they’re] a lot more active [they’re] a lot more smiley [they wake] up smiley but I don’t know whether that’s an impact from the infusion but I definitely see [they want] to get up and run around a lot more and [their] mood generally seems better…. I think those three positives are really massive and there’s not much more I can add to that. [They’re] more happier*,* [they’re] more active and [they’re] sleeping better.”* Parent H, 12 m (RDEB Severe, Placebo/UC-MSCs*)*
Mental well-being	*“…but I also think it’s made a difference to [their] mental wellbeing I think [they’re] bit more positive about things and more willing to be engaging in stuff.”* Parent E, 12 M (RDEB Intermediate, Placebo/UC-MSCs)
Energy	*“Everything really*,* so energy has been fantastic*,* health has been great [they haven’t] stopped”.* Parent B, 3m (RDEB Intermediate, UC-MSCs/Placebo) *“the weird thing is when [they were] getting ill [they have] just been recovering in the same time as [their] brother whereas normally illness affects NAME 1 much more because it causes inflammation so yeah [they’ve] just been able to cope with everything so much better and have lots of energy and I suppose life has felt normal.”* Parent B, 3 m (RDEB Intermediate, UC-MSCs/Placebo)
Eating	*“I think it’s just been lovely to see [them] well like having energy and having an appetite I suppose actually that’s the one thing we’ve noticed as well [that they have] put on weight.”* Parent B, 3 m (RDEB Intermediate, UC-MSCs/Placebo)
Bathing	*“...because [their] wounds are healing quickly so it’s easy to do bath*,* before like I said it’s hard to me to put water in body because its stingy and [they] like stand up in the bath and says it hurt me but now it’s better.”* Parent C, 3 m (RDEB Intermediate, US-MSCs/Placebo)
Daily activities	*“I can like I couldn’t really run run run but I can run run run now ….* *Like I can go out and play with my friends more.”* Patient E, 12 m (RDEB Intermediate, Placebo/UC-MSCs) *“For instance you know school because [they were] in a little bit less pain and less itchy makes things easier for [them] at school just doing daily things but then also sleep because [they're] waking up less to itchy that sort of thing”* Parent F, 12 m (RDEB Intermediate, Placebo/UC-MSCs)

##### Positive impact on relationships

One child reported being able to play more with their sibling, with their parent suggesting it was due to the child being less scared as they were in less pain.

*I think [their] school is the same but when [they're] more playing with [their] siblings now like [they have] a little brother and [they play] with more I think [they're] a little bit less scared maybe because [they're] in less pain” Parent G*,* 12 m (RDEB Intermediate*,* Placebo/UC-MSC)*.

##### Reported negative effects

There were only a few reports of the negative effects of the infusion, with one young person reporting that their heart rate increased immediately after the infusion, and another reporting being extremely tired after the infusion. There were several complaints about the cannula being difficult in younger children. One parent complained of a sweetcorn smell permeating their child and home.

*I don’t think [they’ll] ever get used to it I mean they do put numbing cream on [their] skin before they put the needle in and last time the doctors ended up doing it because their specialist nurses weren’t in to put the cannula in or they were really busy and it took them ten minutes to put it in and [they were] crying the whole time that’s the stuff I really wish be avoided.* Parent H, 12 m (RDEB Severe, Placebo/UC-MSCs).

#### Returning of symptoms following infusion/duration of improvement

Two parents reported the difficulty of dealing with the return of symptoms a few months after the infusion, and there were some comments on the duration of the effect.

*but it just didn’t last long or you know the decline was difficult to deal with and that’s massively challenging for [them] mentally you know to pick [them] back up again and carry on.* Parent F, 12 m (RDEB Intermediate, Placebo/UC-MSCs).

##### No change reported

Nine participants reported no changes after receiving the placebo. The interviewer asked specifically about known symptoms: itch, pain, wounds, eating and toileting as well as asking if there had been any impact on their lives or daily activities. These interviews tended to be shorter, with children in particular giving one- or two-word answers.

*Well no because I didn’t know if there would be any benefit or not*,* whether it’s the placebo or not but yeah I don’t see any difference.* Parent H, 3 m (RDEB Severe, Placebo/UC-MSCs).

Some participants reported mixed results, with some symptoms being positively impacted by the treatment, but other symptoms were not affected. This is reflected in Table 3 below, where the interviews were categorised as ‘Some positive changes’.

*But the skin is still fragile the skin is still taking its time to heal the way it always has [their] mood is better, [they're] more active and [they're] sleeping a lot better*.” Parent H, 12 m (RDEB Severe, Placebo/UC-MSCs).

*I: Ok and what about your itchiness*.

*P: [Participant shakes her head]*

*I: Ok and what about your sleep*.

*P: The same*.

*I: Ok and what about your eyes*.

*P: [Participant shakes her head].* Patient J, 12 m (RDEB Severe, Placebo/UC-MSCs).

##### Unclear results

Some people reported positive results but were unsure if this was related to the infusion or other factors, and there were some interviewees unsure whether things had improved or not as the condition can vary over time.

*but we don’t know if we’re just suggesting because we are in this or because growing up sometimes the fact [they’re] not scratching as much but I don’t know if [they’re] not doing that because of the treatment.* Parent I, 12 m (RDEB Severe, Placebo/UC-MSCs).

##### Comparisons between time points

Thirteen people (8 parents and 5 children) were interviewed at two time points, roughly 3- and 12-months post randomisation (3 months after each infusion) and for ten of these participants there was a clear difference in responses between the two time points, with the interview after the placebo infusions showing no or minimal changes in symptoms or impact on quality of life, and the interview following the UC-MSC infusions reporting positive effects on symptoms or quality of life.

*So I didn’t really notice after the first infusion but on the second infusion I did notice a difference in [NAME 1] skin*,* healing time seemed to be shorter [they] now [seem] to be in less pain after that second infusion and yeah it was a lot more noticeable after the second one so that was a lot more positive experience.* Parent E, 12 m (RDEB Intermediate, Placebo/UC-MSCs).

*The first time was so positive and [their] mood was better his energy was better [they were] a more normal child but since then things have declined and gone back to normal since then and this second infusion didn’t really do much compared to the first one.* Parent B, 12 m (RDEB Intermediate, UC-MSCs/Placebo).

There was an overall feeling that participants thought they knew when they had the active infusion and when they had the control infusion based on their symptoms, but the differences between the interviews varied across participants. There appeared to be a smaller positive effect for children with severe RDEB.

Table 3 provides an overview of each interview as judged by the qualitative researchers (SD and KB) alongside their allocations, age group and RDEB severity; a more detailed version can be found in the main trial report [16]. Across the interviews, there was often impact reported on some symptoms and not others, and it seemed that some symptoms were more salient to the participants than others, but the researchers felt that there was often a clear message coming out of the interviews. Whilst still blind to the treatment the qualitative researchers categorised interviews as: ‘Overall positive’ where there were positive impacts reported on a range of symptoms, ‘Some positive changes’ where they reported positive impacts on a few of their symptoms or quality of life, ‘One small positive change’ where a positive impact on one symptom was reported, ‘Unclear but some positive changes’ where positive changes were reported but the participant claimed to be unsure of the impact of the treatment (e.g. one participant thought it might have been improvement from the weather), or ‘No changes reported’ where participants reported that there had not been improvement on any of their symptoms or quality of life. There are a couple of more nuanced descriptions provided with some further detail where required.

In addition, Table 3 provides the order in which the participants received the placebo and US-MSCs, with participants A-D receiving the active treatment first, placebo second, and participants E-J receiving the placebo first, and the active treatment second. There does not seem to be a difference in responses following the placebo stage dependant on the order of the infusions, with 3/6 of the interviewees that received the active treatment first reporting some improvements at the placebo stage, and 4/10 of the interviewees that received the placebo first reporting some improvements at the placebo stage.

Two of the child participants (D and E), and one parent (A) reported positive effects at both timepoints. One child (E), who received the placebo first, reported positive impacts at both time points, but the parent that was interviewed reported there were no changes at one of the time points. One participant (D), who received the treatment first, reported some positive impacts at both time points but overall thought there was hardly any impact, they also had previous experience of stem cells and thought the impact was not as large as in the previous (uncontrolled) trial.

We could not compare time points for one patient (G) and two parents (D and G). One patient and parent pair (G) both reported positive symptoms after receiving the placebo at the first time point and then withdrew, and one parent (D) reported positive symptoms after receiving the placebo in their 12 months interview but did not have an interview at 3 months. All these interviews were 3 months after they had received placebo, but we do not have data following having the treatment to compare responses.

**Table 3:** Qualitative researchers’ (SD and KB) judgement of overall responses prior to unblinding

ID	Patient details	Order of infusions	Role	UC-MSCs	Placebo
A	Under 6 years* RDEB intermediate	UC-MSC/ Placebo	Parent	Overall positive	Overall positive
B	Under 6 years* RDEB intermediate	UC-MSC/ Placebo	Parent	Overall positive	No changes reported
C	Over 10 years RDEB intermediate	UC-MSC/ Placebo	Patient	Overall positive	No changes reported
			Parent	Overall positive	Compared this time to last time by saying that the 1st infusion was much better.
D	Over 10 years RDEB severe	UC-MSC/ Placebo	Patient	One small positive change	One small positive change
			Parent	Not interviewed	Overall positive
E	Under 10 years RDEB intermediate	Placebo/ UC-MSC	Patient	Overall positive	Some positive changes
			Parent	Overall positive	No changes reported
F	Over 10 years RDEB intermediate	Placebo/ UC-MSC	Patient	Some positive changes	No changes reported
			Parent	Overall positive	No changes reported
G	Over 10 years RDEB intermediate	Placebo/ UC-MSC	Patient	Withdrawn	Some positive changes
			Parent	Withdrawn	Overall positive
H	Under 6 years* RDEB severe	Placebo/ UC-MSC	Parent	Some positive changes	No changes reported
I	Under 6 years* RDEB severe	Placebo/ UC-MSC	Parent	Unclear but some positive changes	No changes reported
J	Under 10 years RDEB severe	Placebo/ UC-MSC	Patient	Some positive changes	Initially reported some positive changes but later in the interview reported none
			Parent	Unclear but some positive changes	No changes reported

*Patients under 6 years were too young to be interviewed

This table has been adapted from the main results paper with permission [16]

##### Willingness to have the treatment again

Parents were asked if they would be willing to let their child have the treatment again and all said they would. There was an overwhelming view that parents would let their child have the treatment if there was even the smallest improvement in their symptoms, because of their view that the improvement in symptoms led to improvement in quality of life. A couple of parents reported that having the cells regularly could lead to a huge impact on their child’ s quality of life, and on their lives.

*I don’t know but I think the first infusion is helped [NAME 1] skin yeah and in my opinion I think you need to be continuing on that treatment otherwise you change the treatment and you find out ok what the reactions to the other treatment and what that means so it’s better everyone says like family members that the feel that this treatment*,* after one months two months I feel the difference and wish we can continue this treatment again.* Parent C, 12 m (RDEB Intermediate, UC-MSCs/Placebo).

##### Comments on the trial

Participants were happy with how the trial was managed and with the staff at the hospitals delivering the interventions. There were comments on the photography being difficult, but people acknowledged that they knew it was important for the trial. The main complaints about the trial were around how long it takes, that some people need to travel quite far to have the treatment and how long the children would have to wait for another dose of the stem cells.

## Discussion

### Summary of main findings

The findings from the Mission EB trial interviews provide insight into perceived benefits of UC-MSCs for children with RDEB and highlight the value of qualitative research in complementing quantitative outcome measures in this population. The quantitative results from the trial showed no change in the main outcome measure, the EBDASI, but there was a reduction in the Instrument for Scoring Clinical Outcomes of Research for EB (iscorEB) clinician subscore and itch. The totality of evidence in the trial showed a positive effect of the treatment in the RDEB-intermediate patients and patients under 10 at 3 months and at 6 months there was a further reduction in itch in all the RDEB-Severe group as well as an improvement in all the IscoreEb outcome scores [16]. The qualitative findings support this. The interviews provided additional evidence of improvements in quality of life that stemmed from improvements to itch and pain symptoms that were not captured in the quantitative outcomes.

#### Beneficial impacts of UC-MSCs

The interviews revealed several positive impacts of the trial treatment on patients’ symptoms and their lives, as reported by both the children and their parents, replicating qualitative findings from a previous study of stem cells in children with RDEB [13]. These benefits included reductions in pain and itchiness, as well as improved wound healing, which in turn led to a reduced need for dressing changes. Improvements in wound healing and pain were also linked to a positive impact on the child’s eating. Furthermore, some parents noted positive effects on their child’s energy levels, sleep and improved recovery from other illnesses following an infusion. These improvements in symptoms often translated into positive impacts on daily activities, with one parent reporting benefits in their child’s mood and energy even when there was no clear effect on RDEB symptoms. Notably, one child reported being able to play more with their sibling, with the parent attributing this to the child being in less pain.

The interviewees reported RDEB to have a huge impact on their lives, in line with previous qualitative research on EB and RDEB [20–22] and the benefits reported were important and seemed significant to the patients and parents interviewed.

The findings showed that three people reported positive impacts at both time points, which complicates the interpretation of the differences between the active and placebo phases for these individuals. This suggests a placebo effect, which is mirrored in the quantitative results. As reported in the main trial paper [16], one of these participants was a parent of a child under one year old (Parent A, Table 3) who received cells in the first crossover period and placebo in the second period. The positive effect reported at the second timepoint could be due to anti-inflammatory benefits lasting for longer periods in this age group. Another child (Patient E, Table 3) who received placebo first, then the active treatment, reported positive changes at both timepoints, where their parent did not, which could relate to response or social desirability bias, where children provide answers they think are expected of them [25]. A patient (Patient D, Table 3) with RDEB severe and was over 10 years old received the active treatment first, placebo second and reported minimal changes at both time points, though they also reported that the impact in this trial was not as large as in the previous uncontrolled trial that they took part in. Additionally, a parent and patient pair that were only interviewed after placebo reported positive effects. These findings could indicate psychological effects of placebo that includes expectation of effect and recall bias and could also be a result being in the trial. There was more intense monitoring of participants during the trial, where wound infections were treated promptly, nutritional status was optimised, and blood deficiencies were corrected through more frequent laboratory assessments which may have contributed to improvements independent of the trial treatment. In addition, although the crossover design provides within-participant comparisons, there may have been other temporal confounds such as natural disease progressions, age development, seasonal effects that have affected the participant reported impact of the infusion.

#### Participants’ ability to discern treatment allocation

A significant finding from the interviews was the apparent ability of many participants, and the researchers, to discern whether they had received the active UC-MSC infusion or the placebo. While the trial was double-blinded, the overall feeling was that participants thought they knew when they had the active infusion and when they had the control infusion based on their symptoms, which was also felt by the interviewer (SD) and the qualitative researcher (KB) during the analysis, which was undertaken blinded. For ten of the thirteen participants interviewed at both time points, there was a clear difference in responses between the two time points that coincided with their treatment in the trial. The fact that participants could often differentiate between the active and placebo infusions based on their symptoms, even when blinded, highlights the potential impact of the UC-MSC treatment on their symptoms and well-being.

#### Comparison of patient perceptions and quantitative outcomes

As reported in the main trial results paper, outcome measures used for EB may not always be sensitive enough to detect the nuances of treatment effects reported by patients and their families, this is often the case for outcomes in rare diseases [19]. These measures are globally recognised to be sub-optimal and an international collaboration was established in 2023 to develop more comprehensive and standardised outcome measures for this heterogeneous disease [17, 18]. The small sample size due to RDEB being a rare disease also meant we did not expect to see statistically significant results in the trial.

EBDASI and IscorEB are not specific scores for RDEB, they cover 4 different types of EB (EB simplex, Dystrophic EB, Junctional EB and Kindler EB), and EBDASI was developed to differentiate different types of EB and monitor in the long-term the disease progression, but not to measure change in a very short period. In addition, these scores were completed at one specific point in time, which in a continuous progressive disease that is affected by multiple factors (like skin infections, weather, nutritional status, etc.) might not reflect symptoms during the 3 month follow up period. The interviewer asked participants about the whole period since receiving the infusion, and the qualitative data may therefore have captured a better overall view of the impact of the treatment.

In addition, children’s expectations and the perspective of their own pain and limitations are different to their parents and will differ due to severity because of their baseline pain or symptoms, i.e. it depends on what they are used to. Children with RDEB severe might not report as much of an impact if they get a bad wound as those with a lower baseline pain or fewer symptoms, or a slight improvement might have more of an impact on their lives than the same improvement for an RDEB intermediate patient.

#### Importance of qualitative research

The findings from this interview study strongly suggest the importance of incorporating qualitative research alongside quantitative measures in clinical trials for conditions like RDEB. RDEB has significant physical, social, and economic consequences, leading to reduced quality of life for patients and their families. Previous qualitative research has also demonstrated the broad impact of EB on the whole family unit [20]. The current study reinforces these findings by capturing the lived experiences of patients and parents participating in the Mission EB trial.

The reported improvements in areas like pain, itch, wound healing, energy levels, and the ability to engage in daily activities and play are valuable outcomes that may not be fully captured by quantitative measures like the EBDASI score.

The qualitative data provides context to the quantitative outcomes of the Mission EB trial. The richness of the interview data allows for a deeper understanding of how the treatment affected the daily lives, routines, and emotional well-being of the participants and their families. The hopes and willingness of parents and children to participate in the trial, even with uncertain expectations, and their reported desire for continued treatment based on perceived benefits further emphasise the significance of these findings. We recommend that trials using quantitative outcomes that might not be sensitive to change, or trials in rare diseases undertake qualitative research to capture the impact of the treatment on participants, and that in crossover trials interviews are conducted at more than one time point.

### Strengths and limitations

#### Strengths

The study employed a qualitative research design using semi-structured interviews, which allowed for an in-depth exploration of the impact of the Mission EB trial treatment on patients’ and parents’ quality of life and patients’ symptoms, capturing their experiences and perspectives in their own words. Interviews were undertaken and led by academic researchers with no commercial interest in the trial. This approach is valuable for understanding the nuanced impacts that quantitative measures might miss and highlighting the potential benefits of UC-MSCs in ways that quantitative measures alone may not capture, for example in this study we identified impact on sleep that was not captured in the quantitative results. As this trial was a crossover trial, interviews were conducted at two time points (3- and 12-month follow-up), providing an opportunity to explore effects at both timepoints and potential changes in experiences and perceptions over time. Including the perspectives of both patients (children and young people over 6 years old) and their parents/guardians offered a more comprehensive understanding of the impact of RDEB and the trial treatment on the whole family. Both the interviewee and interviewer were blind which removed an element of bias which is important here as this treatment gives hope and so there is potential to overstate the effects.

#### Limitations

A key limitation is the small sample size, with interviews conducted with ten parents and six children (thirteen individuals at both time points) and did not account for data saturation due to the limited number of individuals with RDEB. Most of the parent interviews were conducted with mothers (9/10) and this gender imbalance may have introduced a specific parental perspective which differs from fathers’ perspectives. However, even with this relatively small sample size we may have achieved saturation based on a systematic review of sample sizes for data saturation in qualitative studies (Hennick and Kaiser, 2022). This small sample size may limit the generalisability of the findings to the wider RDEB population, though it does include a third of the trial population. We did not ask interviewees to check or provide feedback on the interview transcripts or the analysis, which could have provided an additional layer of validation and ensured the accuracy of the interpretation.

As with any interview-based study, there is a potential for recall bias, where participants may have difficulty accurately remembering past experiences or events. The overarching categorisation of each interview as positive, negative, or no change provided a summary view, but it might have oversimplified the complexity and nuances within individual interviews. Maintaining blinding during analysis was important to minimise bias, but it meant that the analysis did not consider the patient demographics, timepoint or the RDEB severity. RDEB severity appeared to lead to different outcomes in the quantitative data, and so we reflected on that following the analysis, but the qualitative findings might have been strengthened by analysis by sub-type which could help inform future trial design or targeted therapy.

Despite these limitations, the qualitative research provides valuable and rich insights into the lived experiences of children with RDEB and their families participating in the Mission EB trial, complementing and adding to the quantitative findings.

## Conclusion

This qualitative research component of the Mission EB trial provides valuable insights into the perceived benefits of UC-MSC treatment for children with RDEB. The qualitative data regarding symptom improvement and the participants’ and parents’ ability to potentially distinguish the active treatment provide insights into perceived benefits of UC-MSCs for RDEB. It also highlights the importance of qualitative methodologies in validating the trial outcomes and capturing the meaningful impact of interventions on the lives of patients and their families, particularly in conditions where standard quantitative measures may not fully reflect the lived experience, such as RDEB.

## Supplementary Information

Below is the link to the electronic supplementary material.

Supplementary Material 1

## Data Availability

The datasets generated and/or analysed during the current study are not publicly available due to the small RDEB population and the risk to anonymity but may be available from the corresponding author on reasonable request.

## Abbreviations

EB: Epidermolysis Bullosa
EBDASI: Epidermolysis Bullosa Disease Activity and Scarring Index
iscorEB: Instrument for Scoring Clinical Outcomes of Research for EB
Mission EB: Double blind placebo controlled study of mesenchymal intravenous stromal cell infusions in children with Recessive Dystrophic Epidermolysis Bullosa
MSC: Mesenchymal stem cells
RDEB: Recessive Dystrophic Epidermolysis Bullosa
UC-MSC: Umbilical cord tissue-derived mesenchymal stem cells
