# Supplementary material for: Mesenchymal stromal cell infusions of umbilical cord-derived mesenchymal stromal cells in children with Recessive Dystrophic Epidermolysis Bullosa (MissionEB): a qualitative sub study of a randomised, double-blind, placebo controlled, crossover, phase 3 trial
Source: Orphanet J Rare Dis. 2026 Jun 3;21:272. doi: 10.1186/s13023-026-04350-1 (PMC13455256; doi:10.1186/s13023-026-04350-1)
Supplement: Supplementary file 1 [file 13023_2026_4350_MOESM1_ESM.docx]

Supplementary material

Additional file 1- Topic guide

Topic guide for children

Introduction

- Ensure the child has received and read the children’s Patient Information Sheet.
- Explain the aim of the chat today.
- Outline what the meeting will be like (drawing/chat); explain that it will not take longer than 30 mins and if they want to stop this will be ok.
- Explain that if the child does not want to answer a particular question then that is OK
- Explain purpose of digital recording and what happens to the recording you make
- Emphasise that the people who they met for their treatment will not be affected in any way.
- Explain that we will let all children in the study know what we find
- Ensure they are still willing to take part.
- Allow time for any other questions.
- Take child and parent consent.

My name’s [NAME] and I’m a researcher. I work for the University research team, not for the hospital and I am not a nurse or a doctor. My job is to come and ask you questions about some treatment you had, and if it has helped you at all.

Recently you took part in some research that we have called Mission EB. The research is looking at whether your EB can be improved using special cells and you went to hospital for infusion a few months ago. I would like to find out what you think about the treatment, and how you felt before, during and after you went to hospital.

There are no right or wrong answers, we just want to find out what you think. Whatever you say will not affect the people at the hospital or any of your care in hospital in anyway.

To help us with this study, we are going to use this recorder to make a recording of what we all say today. Nobody will be able to identify you from that recording other than me.

Are you happy to continue?

Do you have any questions?

I’m now going to switch on the recorder.

[Switch recorder on]

Confirm assent

Introductions for the recording

*Not all question may need to be asked if the answer has been covered in previous answers.*

*EB background*

How would you describe a typical day in your life?

How does EB affects you the most? Is it the pain, itch, dressing changes, baths, sleep?

*Treatment effects*

How did you feel about going through the treatment overall?

- What were the good things about it?
- Was there anything you didn’t like about it?

How did you feel after you’d had the infusion?

- On the day
- In the days after the treatment

Do you think having the treatment has made a difference to your symptoms?

Has there been an impact on dressing changes or baths? Or on anything else?

Do these activities take the same amount of time as they did before having the infusion?

Have you noticed any difference in pain?

Have you noticed any difference in wound healing?

Has there been any difference in your sleep?

Have you found an impact in your every-day activities?

- Are you able to do things you were not able to do before?
- Have you started any new activities?
- Prompt regarding eating and toileting if not mentioned

Do you think having the treatment has changed your life in any way?

Did you expect these changes?

How long after the treatment do you think any of these changes lasted for?

Did you expect any other changes that did not happen?

Questions for parents

*Not all question may need to be asked if the answer has been covered in previous answers.*

*EB background*

How would you describe a usual day for you and your child? What activities do you usually do?

What is the most complicated symptom? (overall pain/ skin pain/mouth pain/eye pain/bone pain/itch/swallowing difficulties/difficulties having bowel movements or urinating)

What are the daily activities that you find more complicated? (sleeping, eating, drinking, going to school, playing games, drawing, bathing)

Were you looking forward to your child having the treatment?

*Treatment effects*

How did you feel about going through the treatment overall?

- What were the good things about it?
- Was there anything you didn’t like about it?

Would you change anything about it?

- Is there anything that would make the treatment better?

Do you think having the treatment has made a difference to your child’s EB symptoms?

- Has there been an impact during dressing changes or baths? [take less time, are less painful..]
- Have you noticed any difference in the healing of the blisters?
- Has there been a difference in your child’s or your daily activities?
  - Prompt regarding eating and toileting if not mentioned
- What is the most important change that you have noticed?
- Has there been any change in your child’s quality of life?
- Has there been any change in your quality of life?

Did you expect these changes?

Did you expect anything else that did not happen?

Do you think the likely benefits of the treatment are worth the time, energy and effort that needs to be put in?

- If so, why do you think that?
- If no, why do you think that?

If it was up to you, would your child have the treatment again?

How do you think your child or other children with EB would respond to receiving regular stem cell therapy?

Would you be happy to support your child in further stem cell therapy?

Additional file 2 - Coding tree

| **Name** | **Description** |
| --- | --- |
| RDEB symptoms | Codes relating to the impact of RDEB on patients’ symptoms |
| Bathing | Symptoms relating to bathing |
| Bowels | Symptoms relating to toileting |
| Eating | Symptoms relating to eating |
| Itch | Symptoms relating to itching |
| Pain | Symptoms relating to pain |
| Sleeping | Symptoms relating to sleeping |
| Wounds and dressings | Symptoms relating to wounds and dressings |
| Life with RDEB | Codes relating to the impact of RDEB on patients’ lives |
| Activities | Impact on daily activities |
| Social life | Impact on the patients’ social life |
| Energy and mood | Impact on patients’ energy and mood |
| Parents life | Impact on the parents’ lives |
| Routine | Impact on the patients’ and families’ daily routine |
| School | Impact on patients’ school lives |
| Quotes | This code highlighted quotes for use in reports to demonstrate the findings |
| Treatment | Codes relating to the impact of the treatment |
| Expectations of treatment | Patient and parent views on their expectations of the stem cell treatment |
| Experience of treatment | Codes relating to the patient and parents experience of the trial treatment |
| Comparisons between timepoints | Specific comments relating to patient and parent comparisons between the time points from the second interview |
| Dosing | Statements around the dosing schedule in the trial and afterwards |
| Would they have it again | Statements from parents around whether they would want their child to have the treatment again |
| Response to treatment | Codes relating to the response to the trial treatments |
| Immediate response | Statements about any immediate response to the treatment |
| Negative | Statements about any negative impacts of treatment |
| Returning symptoms | Statements around the impact of returning symptoms (following a reported improvement) |
| No change | Statements on the treatment having no impact on symptoms or quality of life |
| Positive* | Statements about any positive impacts of treatment |
| Unclear | Statements around potential impact of treatment, but the patient or parent unclear on whether this was due to the trial treatment, or for another reason (e.g. change in other meds, being on holiday) |
| Trial information | Patient and parent comments on trial information |

*These were also coded to ‘RDEB symptoms’ and ‘Life with RDEB’, making it possible to pull out the response in relation to symptoms and daily living.

RDEB = Recessive Dystrophic Epidermolysis Bullosa

Additional file 3 – Interview length

| Interview ID | Patient or parent | 3-month interview length (mins:secs) | 12-month interview length (mins:secs) |
| --- | --- | --- | --- |
| A | Patient | Too young | Too young |
| A | Parent | 23:19 | 20:55 |
| B | Patient | Too young | Too young |
| B | Parent | 42:36 | 19:11 |
| C | Patient | 20:26 | 06:23 |
| C | Parent | 16:53 | 12:30 |
| D | Patient | 11:05 | 09:58 |
| D | Parent | Unwell | 15:39 |
| E | Patient | 13:48 | 06:32 |
| E | Parent | 32:15 | 15:41 |
| F | Patient | 04:06 | 05:07 |
| F | Parent | 20:50 | 12:50 |
| G | Patient | 10:10 | Withdrew |
| G | Parent | 10:10 | Withdrew |
| H | Patient | Too young | Too young |
| H | Parent | 23:08 | 15:32 |
| I | Patient | Too young | Too young |
| I | Parent | 38:39 | 33:28 |
| J | Patient | 23:34 | 12:11 |
| J | Parent | 15:58 | 09:45 |
